# Toxinologic and Pharmacological Investigation of Venomous Arthropods

**DOI:** 10.3390/toxins14040283

**Published:** 2022-04-15

**Authors:** Gandhi Rádis-Baptista, Katsuhiro Konno

**Affiliations:** 1 Laboratory of Biochemistry and Biotechnology, Institute for Marine Sciences, Federal University of Ceara, Fortaleza 60165-081, Brazil; 2 Institute of Natural Medicine, University of Toyama, 2630 Sugitani, Toyama 930-0194, Japan; kkgon@inm.u-toyama.ac.jp

**Keywords:** arthropod venom, venomics, venom peptides, structure-activity relationship, venom-derived peptide leads, venom-to-drug application

Arthropods comprise the largest group of living animals, including thousands of species that inhabit marine and terrestrial niches in the biosphere. Among the major groups of terrestrial arthropods, several classes contain venomous species, such as arachnids (scorpions and spiders), hymenopterans (ants, bees, and wasps), and chilopods (centipedes). Many have well-developed venom apparatus and rich blends of toxins in their venoms used successfully for self-defense and prey capture [1]. Some of them are harmful to humans, and even today, they cause many poisoning incidents worldwide [2,3,4]. Today, the arthropod venoms are recognized sources of bioactive compounds’ chemical diversity and structural richness and, consequently, possess an unlimited potential for drug discovery and pharmacological application of their components. Arthropod venoms contain peptides and proteins as principal components and small organic molecules (e.g., biogenic amines and polyamines), which may synergistically disrupt the physiological circuit of victims or prey. Thus, the chemical and pharmacological investigation of arthropod venoms has been one of the significant aspects of Toxinology that have made it possible for a prospective molecular pharmaceutical intervention to treat, for example, chronic pain [5], immunological [6,7] and neurological disorders [8,9], and infections caused by multi-drug-resistant microbes [10,11].

Additionally, an exciting application of arthropod venom peptides as environmentally friendly insecticides has been introduced [12,13]. In recent years, the remarkable progress of analytical methods by mass spectroscopy combined with transcriptomic and proteomic approaches and other “omics” methodologies, such as metabolomics, made it possible to reveal the diversity and usefulness of the venom components from some tiny arthropod species [14,15,16,17,18,19]. However, given the vast number of species of arthropods, there are still many understudied venoms that demand more detailed investigation regarding the pharmacological mode of action and structure-activity relationships, aiming at the medical application of native venom components and derivatives.

The present Special Issue continues the previously published Special Issue “Arthropod Venom Components and their Potential Usage” [20] that brings together several articles and original research on this matter. Herein, studies on biological activities, toxicological and pharmacological aspects of isolated venom components or complex crude venoms from various species of arthropods are reported. For instance, in a detailed structural and biological characterization of GTx1-15, an ICK-like peptide from the tarantula spider *Grammostola rosea* venom that inhibits specific subtypes of calcium and potassium ion channels, Kimura [21] demonstrated that GTx1-15 possesses an excellent scaffold amenable for in vitro evolution. GTx1-15 is highly stable to heat and blood serum, and it is an excellent prototype to be converted into non-cytotoxic and non-immunogenic pharmacological agents to modulate ion-channel activity selectively. In another article, Krämer and colleagues [22] investigated linear peptides’ selective antimicrobial, insecticidal and cytotoxic activity from the venom of the pseudoscorpion *Chelifer cancroides.* They demonstrated that the predominant venom peptide checacin1 and three of its less abundant truncated forms are active against aphidians with variable efficacy, but all have the potential to be developed into natural pesticides. Additionally, checacin1 displayed antimicrobial activity against methicillin-resistant *Staphylococcus aureus* with the MIC in the micromolar range despite its cytotoxicity compared to the less bactericide and cytotoxic truncated peptides. Thus, it is argued that tuning the desired effects with low cytotoxicity is possible with these linear peptides from the pseudoscorpion venom.

In a paper by Lopes and coworkers [23], they characterized a sphingomyelinase D (SMase D) from the venom of the spider *Sicarius tropicus.* They showed that the intrinsic features of this venom enzyme differ mechanistically from its counterpart in the venom of the spider *Loxosceles laeta*. The *Sicarius* venom SMase D shares structural and functional features with bacterial sphingomyelinase D rather than with Loxoscele SMase D. Moreover, the authors discussed the distinct toxin profile of venom contents in male and female *S. tropicus* spiders, and other *Sicarius* and *Loxosceles* species, indicating that venom variability in spider venom may have been linked to the level of envenomation.

To identify peptides from the venom of the Scoliid Wasp *Campsomeriella annulata annulatathat*, which paralyzes the prey and nourishes their larvae, Alberto-Silva et al. [14] conducted a comprehensive proteomic analysis using high-resolution liquid chromatography coupled to mass spectrometry and peptide mass fingerprinting of the crude venom. The venom peptides disclosed correspond to two classes: bradykinin-related peptides (e.g., α- and β-campsomerin) and linear α-helical peptides (e.g., annulatin). These peptides showed differential effects on cell viability and histamine-releasing in vitro with negligible hemolytic or cytotoxic effects.

Considering the investigation and characterization of components from crude venom of solitary hymenopterans, an article written by Correia et al. [24] reports the initial toxinologic study of the crude venom from the solitary foraging predatory ant *Ectatomma opaciventre*. This predatory ant species is endemic to the Brazilian Cerrado. The crude venom interferes with the coagulation cascade and hemostasis in vitro, and it is also cytotoxic to lung tumor cells and deleterious to *Leishmania* viability. These findings shed light on the venom of *E. opaciventre* as an exciting source of bioactivities to be pharmacologically tackled. The importance of the toxinologic studies of crude venoms and toxic extracts of arthropods is also exemplified by an article by Moraes and colleagues [25]. They demonstrated the effects of *Lonomia obliqua* caterpillar bristle extract on the human polymorphonuclear neutrophil (PMN) fates. The *L. obliqua* bristle extract induces PMN-mediated pro-inflammatory responses at different molecular levels involving free-radical oxygen species.

Last but not least, Diáz-Navarro and coworkers [26] investigated the antiparasitic activity of extracts prepared from toxic beetles (Tenebrionidae and Meloidae) from Steppe Zones (the inhabiting region of the Great Bustard, *Otis tarda*). Using gas chromatography coupled to mass spectrometry, antharidin and ethyl oleate were the components in the beetle extracts responsible for the biological activity in *Mylabris quadripunctata* meloid and *Tentyria peiroleri*, respectively.

Altogether, these articles make the present Special Issue an additional source of information that illustrates the potential to discover target-specific bioactive molecules and peptides from arthropod venoms, which comprise excellent sources of peptide structures and organics with exclusive intrinsic biological activities. The investigation of isolated venom components and complex mixtures in crude venom and extracts allows the deciphering of biological processes and the conversion of these into prototypes and products for medical and pharmaceutical biotechnology applications now and in the future.
